# The Blood

**Published:** 1888-11-17

**Authors:** 


### PHYSIOLOGY AND ITS USES.

(*Continued from page* 60.)

### THE BLOOD.

MOST people attach some importance to the colour of the hair or to the shape of the nose; but these are mere trifles compared with the quality of the blood. This fluid may be good, bad, or indifferent; but whatever its character, its influence upon its possessor is of the most significant kind. No man is responsible for the blood he is born with, but perhaps every man, by his own voluntary actions, has made his blood better or worse before he has reached forty years of age. The blood is the most fundamental part of a man's organism, and there is no part over the character of which he can exercise more practical control. It is very desirable to get this idea thoroughly worked into the mental constitution if the study of the blood is to be anything more than a matter of merely curious interest. Reading, if there be no intention of making use of the facts and principles set forth, is of the nature of injurious dissipation; and this is quite as true of religious or scientific, as of what is called "light" reading. Perhaps it is more injurious to read scientific books without any practical object in view than to read novels or light essays.

Normal human blood is a thickish red fluid. If any one wants to convince himself of this, let him twist a pocket handkerchief tightly round his finger, leaving about half an inch of the tip uncovered. Now prick the bluish end with a clean needle, not with a pin. In a moment a drop of blood will be available, which will not only show the colour and consistency, but also most of the other important facts which may hereafter be referred to.

The blood, though red, is not of the same degree of redness in every part of the body; some of it is bright red, and some bluish red. The blood which is of a bluish tint is not, as some might suppose, of a superior quality to the other; on the contrary, it is inferior. It is impure, whilst the bright red is pure. The blue-blooded man is a most unhealthy and unwholesome creature. Blue blood, however, may be rapidly changed to red by exposure to fresh air. This is what is going on in the lungs continually. At every stroke of the heart a definite portion of blue blood is exposed in the lungs, which, having been turned into red by the exposure, is rapidly passed on to make room for the inflow which results from the next stroke. An easy way of showing how dark blood may be made bright red by exposure is to take an animal's fresh liver, and cut it in two at the thick part. At the moment of cutting, the surface will be quite dark. In a very short time it will have changed to bright red. A portion of the oxygen of the air has, in the meantime, been absorbed. This process is called oxidation, and the operation is a very easy and convincing way of carrying home to the popular mind the undoubted fact of blood oxidation.

To return to our pricked finger! There is now ready for experiment a pretty large drop of blood, and it is bright red, because having been exposed for some minutes to the air it is well oxidised. Where has it come from? From the capillaries of the skin, little vessels so small that they cannot be seen at all without a high magnifying power. What does it consist of? It consists of—first—a fluid, which, taken by itself, is seen to be neither red nor thick, but colourless and transparent. This is called the *plasma*, or *liquor sanguinis*; secondly—a solid or solids immersed in the fluid. These solids are called *corpuscles*. The word "corpuscle" merely means "little body," and therefore the name does not teach us much. The corpuscles are of two kinds, red and white; though by far the larger number are red. Of the white there are not generally present in healthy blood more than three or four to the thousand. It must not, however, be imagined that because they are so few in number the white corpuscles are comparatively unimportant. On the contrary, they are of marked significance in the economy, as will be seen hereafter.

These are what may be called the broad facts about the constitution of the blood. Every learner should try at the outset to remember that it consists of two obvious parts, a fluid called *plasma*, or *liquor sanguinis*, and innumerable little bodies called *corpuscles*, most of which are red; but a few are without colour, or white. A simple, but highly interesting, method of observing the circulation of the blood is by means of the web of a frog's foot. A live frog must, of course, be used. But let no tender-hearted reader feel the least qualm, there is no necessity for vivisection, or for the infliction of any pain whatever. It is probable that, if Froggy could be made to understand the business, he would offer himself as a lay figure at once to any investigator on whose kind feelings he could thoroughly depend.

"First catch your frog." A microscope will also be necessary, and that nowadays need not cost very much. It will be well to put the subject under an anæsthetic for a minute or two, not to save him from pain, for you are not going to inflict any, but to make him lie still. Then stretch the foot sole uppermost, over the slide, so as to clearly expose the web. Now find the focus and look. It is many years since the writer first made this little observation, and yet he remembers with the most vivid pleasure the interesting and truly wonderful sight. A beautiful meshwork of the tiniest possible tubes, the blood-vessels, appeared; in these the blood was rushing along in what seemed to be perfectly orderly indeed, but very rapid torrents. In the middle of the stream floated innumerable little bodies like coins, or thin circular pieces of cork. They were hurried along by the stream at a tremendous pace, some of them being driven over the top of others, or underneath, or alongside them as if in a race for life. Every now and then one or more would be forced outside the mid-current, and getting pushed to the side of the vessel, would stick fast for the fraction of a moment and lose their places. As the stream hurried from vessel to vessel in the meshwork, always however in a forward direction, there would sometimes be a momentary block at one of the junctions. But almost before there was time to note the event in the mind, the foremost corpuscles would alter their circular shape and become long and narrow, just like a dog wriggling himself through a hole in a fence which is too high for him to leap over. Microscopists reveal to all who care to look, a new world of miracle and beauty—we use the word miracle in its literal and classical signification—but none of their revelations are more calculated to delight the imagination than that of the circulation of the blood in a frog's foot.

(*To be continued.*)

